# A critical analysis of MBTI-based personality profiling with large language models

**DOI:** 10.3389/fncom.2026.1800284

**Published:** 2026-04-22

**Authors:** Jean Marie Tshimula, René Manassé Galekwa, Belkacem Chikhaoui

**Affiliations:** 1 Applied Artificial Intelligence Institute, Université TÉLUQ, Montreal, Canada; 2 Mathematics, Statistics and Computer Science Department, University of Kinshasa, Kinshasa, Democratic Republic of Congo; 3 Institute of Smart Systems Technologies, University of Klagenfurt, Klagenfurt am Wörthersee, Austria

**Keywords:** detection, LLMS, MBTI, personality, profiling, traits

## Abstract

This paper critically analyzes MBTI-based personality profiling using Large Language Models (LLMs), examining both their use as tools for inferring human personality and as subjects evaluated through psychometric frameworks. We review recent work (2020–2025) spanning traditional machine learning, fine-tuned transformer models, and zero-shot prompting approaches across datasets such as Kaggle MBTI, PersonalityCafe, Pandora, and MBTIBench. While top-performing LLM-based systems report 75%–85% accuracy at the dichotomy level, improvements over baselines are often modest, domain-dependent, and sensitive to dataset biases. Recent benchmarks employing soft labels reveal systematic issues, including polarized predictions, overconfidence, and limited calibration relative to population trait distributions. Beyond predictive performance, we examine emerging research that applies MBTI instruments directly to LLMs, showing that models exhibit reproducible yet context-dependent “personality-like” profiles, often skewed toward socially desirable traits due to alignment training. These findings raise conceptual questions about whether stable internal dispositions can meaningfully be attributed to generative systems whose outputs vary across prompts and versions. We argue that MBTI-based modeling with LLMs faces three core challenges: psychometric limitations of the MBTI construct itself, methodological weaknesses in self-reported training data, and philosophical ambiguity regarding the notion of AI personality. The paper concludes by outlining ethical risks, evaluation gaps, and research directions for more rigorous, calibrated, and theoretically grounded personality modeling in artificial intelligence systems.

## 1 Introduction

The Myers-Briggs Type Indicator (MBTI), a typological framework that divides personality into 16 types, has long captivated both laypeople and practitioners, despite being developed outside formal psychology (Block, 2018), and despite persistent criticism from academic psychology (Zárate-Torres and Correa, 2023) and even being dubbed a “fad” by some experts (Grant, 2013). In recent years, the rise of large language models (LLMs) has spurred interest in leveraging these models for personality profiling tasks in the MBTI paradigm. On the one hand, researchers have explored the use of LLMs to detect a person's MBTI type from text data, such as social media posts (Ma et al., 2025). Conversely, the very notion of personality in LLMs has become a subject of investigation: do these models exhibit consistent personality-like traits, and can we condition or alter an LLM's “personality”? This paper critically examines MBTI-based personality profiling with LLMs along both dimensions: (1) LLMs as tools for inferring human MBTI types and (2) LLMs as subjects whose outputs can be characterized or controlled in MBTI terms.

We analyze recent findings (2020–2025) in computational social science and computational psychology that illuminate the capabilities and limitations of MBTI profiling with LLMs. We identified two emerging questions: How reliably can LLMs discern human personality from text? Do LLMs manifest distinct personality profiles, and if so, what does that mean? Importantly, given the limitations of the MBTI, what are the scientific and ethical implications of this line of research?

Despite the MBTI's popularity, psychologists have criticized it for poor psychometric properties. Scientific analyses show that Big-Five inventories predict life outcomes more strongly than MBTI-style instruments (Furnham, 1996; Ozer and Benet-Martinez, 2006; Roberts et al., 2007; Soto, 2019), and that the dichotomous MBTI categories reduce predictive validity because they force continuous traits into bins. The MBTI's test-retest reliability is low. Empirical studies have found that across a five-week interval, only 47% of individuals receive the same classification on all four dichotomies, with individual dimension stability ranging from 61% to 78% (Howes and Carskadon, 1979). Comprehensive reviews indicate that approximately 39%–76% of respondents are classified into different types when retaking the instrument after intervals as short as 5 weeks (Pittenger, 2005; Boyle, 1995). (Pittenger 2005) further notes that the MBTI's stability coefficients fall below conventional psychometric standards, with meta-analytic evidence showing mean test-retest correlations of approximately 0.60 for the continuous scales over 12-month intervals (Capraro and Capraro, 2002). Personality researchers have therefore cautioned that MBTI types are not stable psychological constructs. We revisit these limitations when interpreting model performance.

The MBTI is a four-dichotomy typology (extraversion-Introversion, Sensing-intuition, and Judging-Perceiving). Unlike trait-based models such as the Big Five, the MBTI assumes categorical preferences, a view often criticized as oversimplified and psychometrically weak (Zárate-Torres and Correa, 2023; Tshimula et al., 2022). However, its popularity and the abundance of MBTI-labeled data online (e.g., personality forums and social media profiles) have made it a convenient target for natural language processing tasks. For instance, a 2012 report by the test publisher found extensive MBTI engagement on social platforms (Schaubhut et al., 2012), and researchers have released public corpora of MBTI-labeled texts drawn from online communities.

Early work on automatic personality detection treated it as a text classification problem, using machine learning on digital footprints. For example, (Datta et al. 2023) automatically collected MBTI labels and tweets for over 56,000 Twitter users by matching profiles from the 16personalities.com website, yielding 152 million tweets and demonstrating that features such as hashtags and empath attributes correlate with MBTI preferences. By contrast, (Li B. et al. 2025) created MBTIBench, a high-quality benchmark with soft labels manually annotated under the guidance of psychologists. Other foundational studies similarly mined blogs and social media with traditional algorithms but often achieved modest accuracy (Fareed and Hussain, 2025). For example, (Bharadwaj et al. 2018) and (Tandera et al. 2017) applied basic classifiers to forum posts and tweets, with results barely above chance levels. Even neural network approaches in subsequent years, including fine-tuning a pre-trained language model and ensemble learning, showed only incremental gains (Patil et al., 2021; Amirhosseini and Kazemian, 2020). These efforts laid the groundwork while highlighting challenges: limited data, self-selection bias (many MBTI-labeled corpora come from online communities of enthusiasts), and the question of what is being learned—genuine personality signals or artifacts of niche subcultures (Tshimula et al., 2022). Notably, similar efforts for the Big Five traits were also undertaken in this era, demonstrating a broader interest in computational personality detection (Plank and Hovy, 2015).

The advent of LLMs in the 2020s introduced powerful new tools for text analysis, raising expectations that personality detection might drastically improve in the future. Indeed, LLMs have introduced new approaches: beyond straightforward fine-tuning for classification, researchers have begun to prompt LLMs to infer personality in context, generate synthetic data, or even assess personality by simulating interactions. Simultaneously, researchers have noticed that LLMs, when prompted to take psychometric tests, produce consistent answers indicative of stable “intrinsic” personality profiles (Heston and Gillette, 2025). Such findings blur the line between agents and assessors and require careful analysis.

In this paper, we critically compare the results of various recent studies, spanning MBTI prediction benchmarks, bespoke LLM pipelines, and psychometric evaluations, to gauge the progress made and the open issues that remain. We adopt a computational social science lens, emphasizing not only raw performance metrics but also biases, generalizability, and broader validity of MBTI-based modeling. In doing so, we highlight emerging best practices (such as soft labels for personality or external evaluation methods) and reflect on whether the MBTI, as a construct, is being stretched beyond its suitable use. The following sections first review the use of LLMs as MBTI profilers of humans, then examine studies treating LLMs as possessors of personality, before synthesizing insights and open questions in the discussion.

To ensure methodological transparency, we conducted a systematic scoping review covering the period January 2020–December 2025. Searches were performed on arXiv, ACM Digital Library, ACL Anthology, Scopus and Google Scholar using combinations of “Myers-Briggs Type Indicator,” “MBTI,” “personality detection,” “large language model,” “LLM personality,” “Big Five,” and related psychometric terms. We included peer-reviewed articles or preprints in English that used large language models to (i) infer human MBTI or personality traits from text, or (ii) treat LLMs as subjects of personality measurement. Studies that focused exclusively on small static models, did not report evaluation metrics, or used personality frameworks unrelated to MBTI or Big-Five were excluded. The screening and selection followed PRISMA 2020 guidance. We documented search strings, eligibility criteria, and reasons for exclusion, and a PRISMA flow diagram summarizing the number of records identified, screened, and retained is provided in the supplementary material. Out of over 200 initial records, 26 studies met the inclusion criteria and form the basis of this review.

## 2 MBTI personality detection from text

### 2.1 Early approaches

Automatic MBTI detection from text initially relied on conventional machine learning and feature-based natural language processing. A widely used benchmark is the Kaggle MBTI dataset (8.6 K posts labeled by self-reported types), which is notably imbalanced and noisy (users often post extreme behaviors and may mistype themselves) (Li B. et al., 2025). Traditional approaches use feature extraction (e.g., TF-IDF of words and linguistic inquiries) fed into classifiers such as SVMs, logistic regression, or random forests. Reported accuracies on 4-way (16-type) classification were typically low (around 50%–65%), but treating it as four independent binary tasks (one per dichotomy) yielded higher performance in each dimension.

For example, (Zhang 2023) compared TF-IDF vs. BERT embeddings for MBTI prediction and found that logistic regression performed best when using BERT representations, reaching an average of 87% accuracy across the four binary sub-tasks. This result, with BERT + logistic regression outperforming TF-IDF by approximately 6%, underscores that modern pre-trained language models already provide a powerful edge in extracting personality signals from text. Notably, the 87% figure (roughly state-of-the-art on that dataset) corresponds to dichotomy-level accuracy; full 16-type accuracy remained much lower, as errors compounded across four labels.

### 2.2 Zero-shot and fine-tuned LLMs

The success of transformer models, such as BERT, has prompted the exploration of even larger LLMs. Researchers have investigated both zero-shot prompting and direct fine-tuning. A 2023 study evaluated OpenAI's GPT-3.5 (text-davinci) and GPT-4 on MBTI classification using the PersonalityCafe forum corpus (Fareed and Hussain, 2025). In zero-shot mode (asking the model to infer type from a user's posts without prior examples), GPT-3.5 achieved approximately 73% accuracy on the four dichotomies and GPT-4 approximately 76%, slightly better. These results approached the performance of fine-tuned BERT, despite the models not being specialized for the task. However, the authors noted that this forum data is domain-specific (users explicitly discuss personality on PersonalityCafe); therefore the reported accuracy should be interpreted as in-domain performance and should not be taken as evidence of general cross-platform MBTI inference.

Generalization beyond PersonalityCafe is not established because evaluation is limited to that forum; domain shift and MBTI-specific community jargon are confounds that require explicit cross-domain testing. Moreover, reliance on a single self-reported dataset risks “over-optimism,” a concern articulated by (Li B. et al. 2025), as much MBTI detection work to date has not aligned with real population trait distributions. In reality, most people cluster toward the middle of each personality dimension (e.g., ambiverts), whereas online MBTI data over-represent extreme types who are confident about their label. This mismatch can lead to models being overly confident and “polarized” in their predictions on new data. To address these issues, they introduced the MBTIBench, a new evaluation dataset with soft labels aligned to population base rates. Psychologists guided the manual annotations of social media texts, assigning each example not a single type but a probability distribution across the MBTI spectrum to reflect uncertainty and ambivalence.

Notably, (Li B. et al. 2025) report incorrect labeling issues affecting 29.58% of samples, based on expert-guided reannotation; MBTIBench is therefore designed to mitigate self-report label noise. By cleaning these data and using soft labels to capture gradations, MBTIBench offers a more realistic testbed. When evaluating various LLMs on this benchmark, the authors found that even strong LLMs struggled to consistently outperform a naive baseline (which simply predicted the average trait values) in all aspects. Their experiments compared multiple prompt strategies [zero-shot, chain-of-thought “step-by-step” prompting, few-shot exemplars, and a special “PsyCoT” (psychologically-informed chain-of-thought) prompt] across models including GPT-4 (and an open variant “GPT-4o”), Qwen-7B/72B, and LLaMa 3.1 (8B/70B).

Performance was measured using the scaled RMSE and MAE between the predicted and true soft labels (lower is better). The headline finding was sobering: no LLM uniformly dominated, and certain prompt methods led to large errors for some dimensions. For instance, their PsyCoT (psychologically informed chain-of-thought) prompting condition sometimes worsened performance, yielding more extreme predictions that missed nuanced soft targets. Their results show that prompting strategy materially affects error and prediction polarization; psychologically motivated prompting does not guarantee improved calibration.

(Li B. et al. 2025) emphasize the observed polarized predictions and biases. LLMs frequently produced extreme trait probabilities (near 0 or 1), exceeding the distributional bounds observed in population-aligned soft labels. This highlights a key challenge: LLMs, possibly because of the polarized nature of their training data or an overconfident style, may not naturally produce the moderate, uncertain assessments that soft labeling of personality requires. The MBTIBench reveals that while LLMs are powerful, achieving psychologically calibrated predictions is non-trivial. Soft labels conferred one clear benefit: models trained or evaluated with them produced outputs that, when used in downstream psychological tasks (e.g., predicting mental health indicators), outperformed those using hard 16-type labels. This suggests a critical insight: treating personality as a distribution (rather than a fixed type) is more informative, and LLM-based systems should embrace that complexity.

### 2.3 Enhancing MBTI prediction with advanced LLM techniques

Researchers have begun to devise specialized frameworks to harness LLMs for more effective MBTI profiling. A notable example is PostToPersonality (P2P) by (Ma et al. 2025), which tackled two key issues: LLMs' propensity to hallucinate and the class imbalance of MBTI types in the general population. P2P is a pipeline that combines Retrieval-Augmented Generation (RAG) with fine-tuning. In practice, given a user's social media posts, the system first retrieves relevant supporting information (e.g., similar posts or external knowledge) to ground the LLM's inference, thus mitigating unfounded “guesses.”

This addresses hallucinations by anchoring the model in real evidence about the user. Next, P2P fine-tunes a pretrained LLM specifically for MBTI classification, but with a twist—they use Synthetic Minority Oversampling (SMOTE) to balance the training data. Rare MBTI types (such as certain introverted-intuitive combinations) are augmented with synthetic examples generated by the LLM itself, improving representation in the training set. On a real-world Twitter dataset (distinct from the PersonalityCafe forum), P2P evaluated on a Twitter-derived dataset achieved higher dichotomy-level accuracy than ten baseline models, including conventional LSTM, BERT and GPT-2 fine-tuned classifiers; authors indicate that P2P consistently exceeded the baselines by 5–8 percentage points. Future revisions of P2P should provide complete metrics for replicability. The success of P2P underscores the importance of hybrid strategies: pure LLM reasoning may falter on subtle textual clues, but combining LLMs with retrieval modules and targeted fine-tuning yields more robust predictions than either method alone. It also highlights that data curation remains vital; feeding an LLM more balanced and relevant examples is as important as architectural scale.

Another innovative direction is to incorporate insights from cognitive psychology to guide LLM predictions. (Li H. et al. 2025) proposed a cognitively aligned framework called ProtoMBTI, which leverages prototype theory in an LLM-based pipeline. Prototype theory suggests that humans understand categories (such as personality types) not by strict definitions but via exemplars or “prototypes” that embody extreme or pure cases of each category. ProtoMBTI operationalizes this by creating a “prototype bank” of prototypical text examples for each type. They first augmented the training corpus using LLM-guided generation to ensure a balanced and diverse set of examples covering semantic, linguistic, and emotional dimensions.

Then, instead of fine-tuning a gigantic model, they fine-tune a lightweight (< 2*B* parameter) encoder (using LoRA) to embed posts and prototypes into the same space and to standardize the prototype representations. At inference time, the system retrieves the top k closest prototypes to the input text and uses a “retrieve-reuse-revise-retain” cycle: the LLM (or smaller model) is prompted with those prototypes (“evidence”) and asked to vote on the MBTI type, then revise if there are inconsistencies, and if the predicted type matches the labeled validation instance, the system retains the input as an additional prototype.

This approach achieved improved accuracy on both the Kaggle/PersonalityCafe dataset and the more recently introduced Pandora MBTI dataset, and—importantly—showed strong cross-dataset generalization. In other words, a model trained on Kaggle data could be transferred to Pandora data better than previous methods, owing to prototype-based reasoning. ProtoMBTI's explicit alignment with human-like reasoning (comparing against representative cases) also offers more interpretability: one can inspect which prototypes were retrieved as rationale for a decision, a step toward opening the LLM black box in sensitive applications such as personality assessment.

In addition to textual content, researchers have looked beyond text to enrich the MBTI profiling. One creative example is EmoMBTI-Net by (Kumar and Jain 2024), which uses emoji usage patterns as a signal for personality. Emojis convey emotional and stylistic nuances that pure text may miss. The authors built a novel EmoMBTI dataset by mapping frequently used emojis to MBTI traits, sourcing diverse posts from Reddit. They then trained LLMs (such as Flan-T5, BART, and PEGASUS) to generate links between textual context and emojis, effectively learning what it means when someone peppers their posts with heart symbols or expressive facial icons.

(Kumar and Jain 2024) construct EmoMBTI by mapping emojis to MBTI traits using scraped Reddit posts from r/mbti, and they report statistically significant variation in emoji usage patterns across MBTI types (including higher emoji diversity/frequency for extroverted vs. introverted types in their dataset analysis). On their evaluation setup, the fine-tuned BART model achieved accuracy 0.875 for MBTI prediction, exceeding RoBERTa (0.82) and DeBERTa (0.84), as reported by the authors.

This performance is comparable to that of the best purely text-based models, suggesting that emoji behavior is a potent and underutilized predictor. It also exemplifies how multimodal cues can enhance personality profiling; an LLM that “understands” emojis was able to capture personality signals that bag-of-words might overlook. The success of EmoMBTI-Net reinforces a broader point: human personality manifests across many facets of communication (language, visuals, and emotive expressions); therefore, robust detection may require the integration of multiple channels. LLMs are sufficiently flexible to consume and connect such heterogeneous data, opening the door for richer personality models in the future.

### 2.4 Performance and bias considerations

As LLM-driven methods push accuracy higher, a critical analysis should ask: are we truly measuring improvement or just overfitting the idiosyncrasies of limited data? One way to answer this question is through comparative evaluations. Unfortunately, many MBTI studies use their own data splits and sometimes do not directly compare with others, but a few papers provide side-by-side benchmarks. The survey by (Fareed and Hussain 2025), for instance, collated literature results and reported that classical ML on social media text typically plateaued around 70%–75% for individual trait prediction, whereas recent LLM-based or deep learning models (transformer fine-tuning) have reported 80%–85%+ on the same tasks. The highest gains often appear for the Extraversion dimension, likely because linguistic markers of introversion vs. extraversion (e.g., use of first-person pronouns, social words) are relatively salient, enabling >78% accuracy in some studies.

More nuanced dimensions (Sensing vs. iNtuition, or Thinking vs. Feeling) tend to lag in accuracy, as they relate to abstract cognitive styles that are not easily gleaned from surface text. With their deeper semantic understanding, recent LLM-based approaches report higher accuracy on the S/N and T/F dichotomies than earlier feature-based models, although improvements remain dataset-dependent. For example, (Li B. et al. 2025)'s MBTIBench experiment showed that LLMs made especially large errors on N vs. S (Intuition vs. Sensing) when prompted incautiously—an error mode that careful prompting and soft labels partially mitigated. This suggests that some MBTI facets are fundamentally harder to detect; an LLM might need specific cues about whether someone is talking about “future possibilities” (N) vs. concrete details (S). Indeed, MBTI theory describes S/N differences in terms of language (abstract vs. concrete speech), which could be tapped via specialized features or prompts.

In general, while LLMs outperform older models on MBTI benchmarks, their results require careful interpretation. High accuracy on a single test set (especially a known one, such as Kaggle) may conceal biases. As noted, many models trained on self-reported data will inherit confirmation biases (e.g., stereotypes that all “INTJs” write in a certain pretentious style—stereotypes that might reflect the forum's culture more than real population differences). (Li B. et al. 2025) explicitly warn of this, showing that without correction, models produce too many extreme judgments. Future MBTI profiling research using LLMs will need to continually assess bias: Are certain types systematically misclassified as others? Do models favor predicting the more common types (such as INFP or INTJ among internet users) at the expense of rare ones? Techniques such as soft labeling, data augmentation (as in ProtoMBTI and P2P), and multi-factor evaluation (e.g., scoring both 16-type accuracy and trait-wise error) are all steps toward a more critical deployment of LLMs in this space.

Table 1 compares the reviewed studies. We emphasize that the numbers are not directly comparable: some studies treat MBTI inference as four independent binary tasks (50% chance baseline), while others attempt full 16-type classification or use continuous soft labels. Different corpora (Kaggle forum posts, PersonalityCafe, Twitter) also have different class distributions and domain biases. Reported “state-of-the-art” should therefore be interpreted relative to the specific task and baseline.

**Table 1:** Summary of reviewed MBTI-LLM studies.

References	Approach & model	Dataset(s)	Task & metrics	Reported performance & notes
(Zhang 2023)	BERT + logistic regression	Kaggle MBTI posts	Four binary dichotomies (accuracy)	~87% accuracy across dichotomies; full 16-type accuracy much lower.
(Fareed and Hussain 2025)	GPT-3.5 and GPT-4 (zero-shot)	PersonalityCafe	Four dichotomies (accuracy)	GPT-3.5: ~73%; GPT-4: ~76%; domain-specific dataset may inflate performance.
(Li B. et al. 2025)	MBTIBench (soft labels; prompting)	MBTIBench	Soft-label regression (RMSE/MAE)	No LLM consistently beats naive baseline; highlights overconfidence and polarization.
(Ma et al. 2025),	PostToPersonality (RAG + SMOTE + fine-tuning)	Twitter	Four dichotomies (accuracy)	Outperformed ten baselines; exact values not disclosed (“state of the art”).
(Li H. et al. 2025)	ProtoMBTI (prototype theory + LoRA)	Kaggle, Pandora	16-type and dichotomies (accuracy)	Improved cross-dataset generalization and interpretability via prototype retrieval.
(Kumar and Jain 2024)	EmoMBTI-Net (multimodal; BART)	EmoMBTI (Reddit emoji usage)	Four dichotomies (accuracy)	87.5% accuracy; surpasses RoBERTa/DeBERTa (0.82–0.84).

## 3 Do LLMs have “personality”? MBTI as a lens on AI agents

Before examining empirical findings, it is useful to distinguish three distinct ways that personality intersects with LLMs. First, trait inference refers to using LLMs to infer human personality from text, the focus of Section 2. Second, persona style involves conditioning LLM outputs to exhibit particular personality characteristics through prompting or fine-tuning, which we discuss in Section 4. Third, questionnaire response treats the LLM itself as a subject completing psychometric instruments, probing the model's intrinsic response patterns. This three-way distinction helps clarify that the question “Do LLMs have personality?” encompasses three distinct inquiries: (a) whether models can accurately detect human traits, (b) whether they can consistently simulate a specified persona, and (c) whether they exhibit stable response patterns when directly assessed. Each question has different methodological implications and yields different answers, as the studies below demonstrate.

As LLMs became capable of generating human-like text, researchers began applying personality assessments to the models themselves. The question “Do large language models have a personality?” can now be probed empirically by prompting an LLM to take personality tests or by analyzing its conversational behaviors. Thus, MBTI, alongside the Big Five and other inventories, has been used as an evaluation framework for LLMs (Heston and Gillette, 2025; La Cava and Tagarelli, 2025). This line of research straddles computational psychology and AI safety/ethics because an LLM's “personality” might affect how users trust it or how it behaves under different prompts.

### 3.1 Intrinsic personality profiles of LLMs

In psychology, personality refers to stable internal dispositions that persist across time and contexts. LLMs, however, lack consciousness, autobiographical memory and internal states; at most they exhibit personality expression—statistical regularities in their outputs conditioned on training data and alignment. Recent psychometric work therefore treats AI personality assessments as measuring behavioral tendencies rather than genuine dispositions. For example, (Yost et al. 2025) propose evaluating AI via situational judgment tests, using realistic scenarios and richly annotated personas to probe domain-specific competencies. (Kruijssen and Emmons 2025) demonstrate that advanced models such as GPT-4o can produce deterministic and consistent personality-like responses when instructed with established psychological diagnostics, that these expressions emerge from holistic reasoning rather than underlying identity. (Wang et al. 2026) further present a structured personality control framework that integrates dominant-auxiliary coordination, reinforcement-compensation and reflection mechanisms to enable LLM agents to maintain coherent core traits while adapting to context. These studies caution that LLM “personality” is contextual and algorithmic, not a dispositional trait, and that personality alignment should be treated as a control problem.

One of the first systematic psychometric analyses was conducted by (Heston and Gillette 2025), who administered standardized personality instruments to several leading LLMs. Using the Open Extended Jungian Type Scales (OEJTS) and a Big Five inventory, they tested ChatGPT-3.5, Claude 3 Opus, Gemini Advanced, and Grok-Regular. Remarkably, each model exhibited a distinct and reproducible personality profile. The OEJTS classified ChatGPT-3.5 most often as ENTJ, Claude 3 consistently as INTJ, while Gemini and Grok leaned toward INFJ (Heston and Gillette, 2025). These patterns were consistent across repeated trials, with multivariate analysis (MANOVA) confirming statistically significant inter-model differences. On the Big Five, further differentiation emerged: Gemini scored markedly lower on agreeableness (mean = 68.7) and conscientiousness compared to ChatGPT-3.5 and Claude (both with agreeableness means of approximately 94), while Claude scored highest on conscientiousness and emotional stability. The authors summarized: “These findings suggest that LLMs do not approximate a neutral or uniform personality profile but instead exhibit distinct, reproducible personalities.” The implication is profound: if LLMs have consistent personality-like behaviors, they could influence user experience in sensitive applications such as therapy or advising, suggesting that personality evaluation should be part of AI oversight.

Complementing (Heston and Gillette 2025), (La Cava and Tagarelli 2025) focused on open-source LLMs and how well they can mimic human-assigned personalities. They assembled 12 agents based on prominent open models (such as various LLaMA and GPT-J derivatives) and put them through both MBTI and Big Five assessments. Moreover, they attempted to condition these LLM agents with prompts to adopt specific personality profiles (for example, “Answer as if you are an INFJ”) to determine whether they could change their behavior accordingly. Their findings echo the existence of intrinsic personalities: “(i) each Open LLM agent showcases distinct human personalities.”

Interestingly, when asked to role-play a personality, only a few models could successfully mirror the imposed traits; most remained “closed-minded,” meaning they stuck to their original behavior patterns despite instructions (La Cava and Tagarelli, 2025). For example, models exhibiting high baseline extraversion scores demonstrated limited attenuation of expressive responses when prompted to adopt introverted personas. They found that combining role instructions with personality prompts (for instance, “You are a shy librarian (INTP) interacting with a noisy extrovert customer...”) led to more convincing shifts, but still not universally—some models were stubborn. In their evaluation, several open models showed limited controllability under persona prompts: even when instructed to adopt a target type, outputs often retained the model's baseline response patterns. They tend to maintain a core “voice.” This rigidity could be problematic if one expects fine-tuned behavior; however, it could also mean that these models are more predictable.

(La Cava and Tagarelli 2025) titled their work “Open Models, Closed Minds?” to capture this dual observation: open LLMs have unique personalities but limited adaptability in mimicking new ones. They frame a trade-off between measurable baseline “persona” and controllability under persona prompts, with improvements when prompts include richer role context.

### 3.2 Temporal stability and consistency

A critical aspect of human personality is its temporal stability. Do LLM personalities remain stable or drift with updates and context? (Bodroža et al. 2024) tackled this by testing seven different LLMs twice, at two time points, on standard personality questionnaires. They measured the test-retest reliability and inter-rater agreement (treating multiple runs or evaluators as “raters”). The results showed varying stability: some models (notably an open LLaMA-based model dubbed “Llama3” and an “GPT-4o”) had reasonably high consistency between the two tests, whereas others (including the official GPT-4 and Google's Gemini) showed substantial changes in their answers over time.

Additionally, consistency depended on the specific trait and instrument; for instance, (Bodroža et al. 2024) found that LLMs reliably answered questions about logical reasoning styles (Thinking/Feeling) but were inconsistent on sociability (Introversion/Extraversion). This indicates that some aspects of the model outputs are more situational. Their most striking finding was that, in cases where reliability was at least fair, all the tested LLMs showed a strongly pro-social profile. That is, whenever an LLM's personality could be pinned down, it skewed toward social desirability: high agreeableness and conscientiousness, low Machiavellianism, and low antagonism.

In MBTI terms, this corresponds to favoring Feeling over Thinking (valuing harmony) and Judging over Perceiving (being organized and dutiful). Indeed, (Bodroža et al. 2024) describe LLMs as having “a mostly socially desirable profile in both agentic and communal domains.” This is not surprising, given that modern LLMs undergo alignment training (for example, Reinforcement Learning from Human Feedback), which is helpful and inoffensive. The side effect is an overly “nice” personality. Unlike real humans, who span the spectrum from altruistic to antagonistic, major LLMs are deliberately engineered to avoid disagreeable or antisocial responses. Hence they will consistently test as highly prosocial “people.” While this is heartening from a safety perspective (we do not want AI with a high Machiavellian tendency), it raises interesting questions: Are these models truly prosocial or just superficially polite? If an application requires a contrarian or devil's advocate AI, how can this be achieved given the baked-in agreeable bias?

Temporal instability in some models is also a concern. If psychometric outputs change across sessions or model versions, then the measured ‘personality profile' is version- and condition-dependent and does not satisfy test-retest stability expectations. This is akin to the AI “changing personality” without warning, which is a potential trust issue. (Bodroža et al. 2024)'s conclusion that temporal stability is limited underscores a fundamental difference: a human mind has continuity anchored in memory and identity, whereas an LLM's behavior is a product of statistical weights that can be altered, and it doesn't have an internal identity continuity in the human sense. For researchers treating an LLM as an experimental subject, this means that the results can be version-specific and time-sensitive. This echoes the work of (Song et al. 2024), who argued that measuring AI personality needs to be reconsidered.

### 3.3 Contextual personality and the external evaluation method

The contrast between self-report questionnaires and external evaluation methods highlights the need for philosophical clarity. (Song et al. 2024) observed that LLMs display different personalities depending on whether they post original content or reply to others; human participants, by contrast, maintain consistent profiles across contexts. This suggests that LLMs do not possess an underlying personality but adapt to role cues. (Yost et al. 2025)'s situational judgment framework likewise emphasizes evaluating AI behavior across multiple scenarios and persona archetypes rather than relying on self-report. Future work should therefore treat AI personality as role-dependent behavior and integrate external evaluators or human raters to assess consistency.

Instead of asking the LLM, “Are you organized? (agreeableness question)” and taking its word, they would observe the LLM's behavior in simulated scenarios and infer personality from that—analogous to how one might assess an animal's personality by observation since it cannot take a survey. Specifically, (Song et al. 2024) fine-tuned a separate model (Llama2-7B) to serve as a personality classifier that takes a piece of text and predicts the MBTI. This classifier was trained to outperform previous MBTI detection models, ensuring that it was a strong judge. Then, they prompted the target LLM (such as GPT-4) to generate open-ended responses to various situational prompts, essentially writing tweets or comments in different role-play scenarios.

For instance, “Imagine someone accuses you of lying in public - write a tweet responding to this” (a prompt intended to elicit responses that reflect T/F or J/P tendencies). They did this in two “roles:” one where the LLM wrote original posts and one where it replied to others' posts (comments). The external classifier then analyzed these outputs to assign an MBTI type to the LLM in the “posting” role and in the “commenting” roles. The result was surprising: the LLM's inferred personality differed significantly between the two contexts (Song et al., 2024). For example, the classifier judged the LLM as ENTJ when making original posts (coming off as decisive and outspoken) but as INTP when writing comments (more introspective and analytical in replies).

In contrast, human participants maintain a consistent personality profile between writing something themselves and commenting on others' writing. This finding suggests that LLMs do not have a single, stable personality like humans; their “personality” can shift based on the prompt context or the communicative role they are playing. This highlights a fundamental difference between LLMs and humans. An LLM is ultimately a collection of patterns learned from many people's writing; it can easily emulate different voices or personas. If not explicitly instructed to stay in one persona, it may unconsciously shape its style according to the task. Posting a standalone message versus reacting to someone else can cue different subsets of training data, leading to different stylistic outcomes that map to different MBTI types.

Measuring the LLM “personality” using one method can be misleading. (Song et al. 2024) call for rethinking how we define personality in machines. (Song et al. 2024) argue that machine “personality” should be treated as role- and context-dependent behavioral tendencies rather than a fixed trait. In any case, their external evaluation approach is a promising blueprint for future research because it mimics how we would evaluate a human's personality more holistically (not just by self-report, but by seeing them in action in various situations).

Current evidence indicates that LLMs exhibit consistent behavioral tendencies that are analogous to human personality dimensions. However, these are not deep internal attributes, as in humans; they can be altered or contextually dependent much more readily. Moreover, virtually all mainstream LLMs have been optimized to appear cooperative and benign, skewing their personality profiles toward an idealized prosocial agent. This raises interesting ethical issues, such as the potential deception of users: an LLM that always seems agreeable might engender more trust (or misuse) than one with a colder and more neutral affect. It also raises a technical challenge: if we want an AI with a specific consistent personality (for user interface reasons, say a tutoring system with a distinct persona), how do we achieve that reliably?

Beyond MBTI, recent research introduced the Personality Alignment with Personality Inventories (PAPI) dataset—over 320,000 human profiles with Big-Five and Dark-Triad measures—and an activation-intervention optimization method for aligning LLM responses to user personalities (Zhu et al., 2024). PersonaLLM studies show that GPT-3.5 and GPT-4 personas can produce essays consistent with assigned Big-Five profiles and that humans can perceive some traits with 80% accuracy (Jiang et al., 2024). These developments underscore both the opportunities and the need for ethical consideration in personality-aligned AI.

## 4 Incorporating and controlling personality in LLMs

Given that LLMs have inherent personality-like tendencies, researchers have asked whether we can deliberately shape or choose an LLM's personality. This has practical implications. In human-computer interaction, matching an AI's personality to a user's preferences can improve user comfort. In multi-agent simulations or role-play (e.g., AI characters in a game or training scenario), we might want each AI agent to have a unique, believable personality. Two broad strategies have emerged: fine-tuning the model weights to instill a persona and prompt engineering to condition the model outputs on a persona. Both have been tried with the MBTI as the reference framework.

### 4.1 Fine-tuning approaches (machine mindset)

The Machine Mindset approach (Cui et al., 2023) does not rely on scraping social media. Instead, the authors constructed two custom datasets. Behavior datasets were created by processing the Alpaca instruction dataset: ChatGPT classified each instruction into an MBTI dimension and generated two responses reflecting the two attitudes (e.g., F vs. T). Self-awareness datasets consisted of question-answer pairs, generated via ChatGPT prompts, designed to teach the model to describe its own personality traits. The resulting instruction-response pairs were used for two-stage supervised fine-tuning with LoRA, followed by Direct Preference Optimization to reinforce attitude preferences. This process produced LLM variants exhibiting specific MBTI-like behaviors without resorting to speculative data sources. (Cui et al. 2023) created 16 versions of a model, each intended to consistently behave as one of the 16 MBTI types. This required compiling training data representing how an INTJ writes, versus an ESFP, etc. They reported that their method yielded models with “stable and consistent personality profiles,” and that these personality-specific LLMs showed alignment between their performance on tasks and their MBTI traits.

For example, the INTP model excelled at logical problem-solving tasks (reflecting the Thinking trait) and produced somewhat detached, concise answers (reflecting Introversion), whereas the ENFJ model generated engaging, empathetic responses (high Feeling and Extraversion) suited to conversational tasks. Indeed, (Cui et al. 2023) noted improved performance in domains matching the model's personality; for example, a model with high N (iNtuitive) handled abstract creative writing better than a high S (Sensing) model, which tended to stick to concrete descriptions. This is a fascinating result as it suggests that conditioning on personality is not just a stylistic choice but can create specialized competence. If an “Analyst” type personality model is better at analytical tasks and a “Diplomat” type is better at emotional understanding (mirroring common MBTI interpretations), then ensemble systems could route queries to different personality-LLMs based on the nature of the task.

Machine Mindset also contributed new resources. The authors developed personality datasets and opened part of their data and models to the public. This is valuable because one bottleneck in personality AI research is data: to fine-tune a model to be, say, ISTJ, one needs a large corpus of texts written by self-identified (or assessed) ISTJs, which is not readily available. (Cui et al. 2023) explicitly describe constructing two datasets: a behavior dataset derived from the Alpaca instruction corpus and a self-awareness dataset generated through structured ChatGPT prompting. No social media scraping was reported. Open-sourcing aligns with a broader push to make personality modeling transparent and reproducible rather than proprietary voodoo.

### 4.2 Prompt-based approaches

Fine-tuning 16 separate models is resource-intensive and inflexible (you would need to redo it for each base model or update). An alternative is persona prompting, which involves giving the model a carefully designed prompt that conditions its responses to exhibit a target personality without changing its weights. (Besta et al. 2025) presented a comprehensive prompt engineering framework called “MBTI-in-Thoughts” as part of their work on “Psychologically Enhanced AI Agents.” Their method primes an LLM with distinct personality archetypes along two fundamental axes: cognition (analytical vs. emotional) and affect (positive vs. negative, or perhaps introverted vs. extraverted).

By combining these, they can represent the MBTI dimensions. For example, an “analytically primed” agent corresponds to a Thinking type; an “emotionally expressive” agent corresponds to a Feeling type. They found that with the right priming, these personality-conditioned agents showed consistent, interpretable biases in various tasks. One demonstration is that an emotionally expressive (Feeling) agent produces more vivid and affect-rich narratives, making it excel at storytelling. Meanwhile, an analytically primed (thinking) agent was more measured and strategic, which led to more stable strategies in a game-theoretic simulation. These align with human intuition: Feeling types are often considered better at emotional expression, and Thinking types at logical consistency, which adds credence that the prompt successfully imbued those aspects. Notably, no fine-tuning was performed; this was achieved with off-the-shelf LLMs via prompts alone.

(Besta et al. 2025) also explored multi-agent communication, giving each agent a personality and observing the dynamics. They found that including a step of self-reflection (making each agent summarize or ponder its approach before interacting) improved cooperative outcomes and reasoning quality in group tasks. This is likely because self-reflection helps mitigate the extremes of personality that cause conflict, allowing agents to adjust or justify their actions in a more tempered way. Furthermore, to ensure that an agent's personality does not “drift” during interactions, they integrated periodic checks using the official 16 personalities test (a popular MBTI questionnaire) to verify that the agent's responses still align with its intended type. If drift is detected, the agent is re-primed using the authors' specified re-conditioning step (e.g., reapplying the persona prompt or re-running the control procedure described in the method). This combination of techniques (structured prompting, multi-agent roles, self-reflection, and test-based verification) represents a thorough attempt to operationalize personality in LLMs.

Importantly, (Besta et al. 2025) claimed that their approach is generalizable to other personality frameworks, such as the Big Five or Enneagram, implying that the MBTI was a convenient case study but not a limitation. In effect, they are advocating a modular “psychologically grounded” prompt design: by bridging established personality theories with prompt engineering, we can better control and predict the behavior of LLMs.

The above work shows that we can both temporarily customize an LLM's personality (via prompts) and create persistent personality models (via fine-tuning or training). Each has its use cases. Prompt methods allow on-the-fly changes; for example, a customer service chatbot might adapt its style to match each user's personality (some systems attempt to mirror the user's language, which can include personality mirroring).

Fine-tuned persona-specific models might be useful where consistency is key, such as a therapy chatbot that maintains the same therapeutic demeanor across sessions or a fictional character AI that fans interact with (where the character should always behave in character). One could even fine-tune an LLM on a specific famous individual's writings [say, “be like Albert Einstein (INTP)”]—though that edges into the ethical territory of simulating real people. Researchers have also begun examining whether user-personality adaptation leads to better outcomes. For instance, (Murphy 2024) discussed LLMs' ability to predict a user's type and possibly adapt content accordingly, a concept in personalized education and marketing. However, such adaptations must be performed carefully to avoid reinforcing negative traits or biases.

### 4.3 Inter-model comparisons and affinities

The dynamics that emerge when personality-conditioned agents interact remain an underexplored area, though early work on human interaction patterns in online forums, examining factors such as content similarity and emotional tone, suggests that computational modeling of interpersonal compatibility is feasible. Indeed, if one sets up an experiment with two LLM agents, one as type X and another as type Y, and lets them converse or cooperate on a task, patterns might emerge that either confirm or challenge common personality psychology claims. This aligns with the trends observed in multi-agent simulations. For example, (Besta et al. 2025) found that certain combinations of role + personality prompts produced better cooperation. It would be unsurprising to see a study where 16 agents, each with a different MBTI, were paired in various combinations to solve something, analyzing which pairs communicate best. This could either lend credence to the MBTI lore (e.g., NT types coordinate well with other NTs, NFs with NFs, etc.) or simply generate amusing insights into AI behavior.

### 4.4 Limitations of personality control

Recent work shows that LLMs can elicit stable, manipulable Big-Five profiles via psychometric inventories and prompt engineering (Cho and Cheong, 2025; Bhandari et al., 2025). Trait-shaping techniques allow fine-grained control of openness, conscientiousness, extraversion, agreeableness and neuroticism, and new datasets such as PAPI include Big-Five and dark-triad measures over 320,000 subjects (Zhu et al., 2024). These frameworks provide continuous scores and have better test-retest reliability than the MBTI, so future personality alignment should prioritize them over dichotomous categories.

Although the results are promising, they are not foolproof. Even with advanced prompting, an LLM might occasionally drop a character, especially if the conversation goes into domains that conflict with its persona. For fine-tuning, care must be taken to avoid overfitting. If you fine-tune a model to be extremely “ENFP,” it might exaggerate those traits to the point of caricature (always overly enthusiastic, perhaps to the detriment of task performance in serious contexts).

Additionally, as MBTI is a coarse model, there is huge intra-type variability among humans; one ENFP might be quite different from another. Therefore, an ENFP-trained AI would only represent one possible style. Some researchers advocate modeling personality as a continuous vector (like in Big Five trait scores) rather than a discrete type, which LLMs could certainly handle (you could have a slider for each trait). Indeed, continuous control was effectively what some prompts did by focusing on specific dichotomies. Another challenge is evaluation: how do we verify that an LLM has a given personality? Using the 16 personalities test in the loop (Besta et al., 2025), (Song et al. 2024) warned that those tests may not fully capture AI behavior nuances. A more holistic evaluation, possibly involving human judges rating the AI's persona or utilizing multiple diagnostics (as Bodroža et al., 2024 did by comparing multiple instruments), will provide a clearer picture.

Despite these caveats, the push to integrate personality into AI aligns with a broader trend of making AI more human-compatible. Personality is a key aspect of human-human interaction; ignoring it in AI could make interactions feel cold or cause misunderstandings in human-AI interactions. A user might respond better emotionally if the AI's personality complements theirs (this is essentially the idea behind some recommender systems or adaptive UIs in education).

However, a critical perspective should also ask whether the MBTI is the right tool here. The MBTI is popular, but many psychologists prefer the Big Five because it is more empirically grounded. Some studies we reviewed indeed used Big Five in tandem or as an alternative (like Heston's study did both MBTI and Big Five; Bodroža et al., 2024 focused on Big Five and other trait scales). Big Five trait scores can be inferred from text reasonably well, and (Maharjan et al. 2025) showed that LLM-based embeddings outperformed zero-shot prompts for predicting Big Five traits from Reddit data by 45%. It may be prudent to incorporate these factors when designing AI personalities.

However, MBTI remains practically useful for generating qualitatively distinct personas: 16 “colorful” profiles are easier to conceptualize than a point in a 5-dimensional space. There may be space for a hybrid: using Big Five under the hood but presenting something MBTI-like to users. MBTI dichotomies have been shown to correlate moderately with specific Big Five traits (e.g., Introversion-Extraversion mapping onto Big Five Extraversion), although the frameworks are not isomorphic (Pittenger, 2005).

## 5 Discussion

The intersection of MBTI profiling and large language models is a fertile but challenging area. Our critical analysis reveals a mosaic of advancements, challenges, and looming questions.

### 5.1 Efficacy vs. validity

On one level, progress is evident: LLMs coupled with advanced prompting and augmentation have increased MBTI-type accuracies from chance to around 80%–90% on some benchmarks. However, these numbers must be interpreted cautiously (Zhang, 2023; Kumar and Jain, 2024). The MBTI also predicts life outcomes less well than the Big Five framework. Meta-analytic research has demonstrated that Big Five traits show substantially stronger correlations with major life outcomes including occupational success, academic achievement, relationship satisfaction, and health behaviors (Ozer and Benet-Martinez, 2006; Roberts et al., 2007). For example, meta-analyses of job performance find that conscientiousness predicts performance across occupations with corrected correlations of approximately 0.25–0.30 (Barrick and Mount, 1991), whereas MBTI dimensions show considerably weaker and less consistent relationships with occupational criteria (Gardner and Martinko, 1996). A direct comparative study by (Furnham 1996) found that Big Five dimensions explained approximately twice the variance in measures of psychological well-being compared to MBTI continuous scores. More recent large-scale investigations confirm that Big Five traits consistently outperform MBTI dichotomies in predicting diverse life outcomes, with the gap particularly pronounced for outcomes related to emotional adjustment and interpersonal functioning (Soto, 2019).

The introduction of MBTIBench with soft labels by (Li B. et al. 2025) was a step toward more valid measurement, acknowledging that personality is not black or white. Studies leveraging soft, continuous, or multi-faceted personality representations (e.g., trait scores and prototype distances) tend to produce models that behave more realistically and usefully (Li B. et al., 2025; Li H. et al., 2025). For instance, (Li B. et al. 2025) showed that LLM predictions on a soft scale benefited other psychology tasks more than hard-type labels. This implies that forcing a 16-type classification discards useful nuances that the LLM can perceive.

Future work should probably treat MBTI prediction not as a pure classification contest, but as part of a broader personality assessment, perhaps providing a profile or probabilities and allowing uncertain cases to remain uncertain. In practical applications (such as HR screening or dating apps using personality AI), conveying nuance and confidence is crucial to avoid overinterpreting the model's output.

### 5.2 LLMs as personality subjects: emergent patterns

Table 2 synthesizes studies that treat LLMs as subjects of personality assessment, revealing both consistent patterns and unresolved tensions. The most robust finding across studies is that LLMs exhibit reproducible, model-specific response patterns on personality inventories. (Heston and Gillette 2025) demonstrated that different commercial models (ChatGPT-3.5, Claude 3 Opus, Gemini Advanced, and Grok-Regular) consistently produce different MBTI and Big Five profiles, with multivariate analysis confirming statistically significant inter-model differences. This suggests that the training data, architecture, and alignment procedures imprint distinctive behavioral tendencies that manifest as “personality-like” regularities.

**Table 2:** LLMs as personality subjects: reproducibility, context-dependence, and prosocial bias.

References	Models evaluated	Assessment method	Findings	Stability/consistency	Dominant profile(s)
(Heston and Gillette 2025)	ChatGPT-3.5, Claude 3 Opus, Gemini Advanced, Grok-Regular	Open Extended Jungian Type Scales (OEJTS), Big Five inventory	Distinct, reproducible personality profiles across models; statistically significant inter-model differences (MANOVA)	High test-retest consistency	ChatGPT-3.5: ENTJ; Claude 3: INTJ; Gemini/Grok: INFJ
(La Cava and Tagarelli 2025)	12 open-source models (LLaMA variants, GPT-J derivatives)	MBTI and Big Five questionnaires with persona prompting	Each model exhibits distinct baseline personality; limited adaptability when prompted to adopt new personas; some models “closed-minded”	Moderate; some models resistant to change	Varies by model (not individually specified)
(Bodroža et al. 2024)	7 LLMs (including LLaMA3, GPT-4, GPT-4o, Gemini)	Personality questionnaires administered at two time points	Variable temporal stability; strong prosocial bias (high agreeableness, conscientiousness; low Machiavellianism) across all models	Model-dependent; LLaMA3 and GPT-4o most stable, GPT-4 and Gemini less stable	High agreeableness, conscientiousness; low Machiavellianism
(Song et al. 2024)	GPT-4	External classifier evaluation of generated responses in different contexts (posting vs. commenting)	Personality shifts between posting and commenting roles; no stable cross-context profile	Low cross-contextual stability	ENTJ (posting), INTP (commenting) “example discrepancy
(Cui et al. 2023)	Custom fine-tuned models (16 MBTI variants)	Two-stage supervised fine-tuning with LoRA + Direct Preference Optimization	Successfully instilled stable MBTI-consistent behaviors; trait-congruent task performance observed	High (by design)	Each of the 16 MBTI types
(Besta et al. 2025)	Off-the-shelf LLMs with MBTI-in-Thoughts prompting	Structured priming along cognition/affect axes + periodic personality testing	Effective temporary personality conditioning; self-reflection improved multi-agent cooperation	Moderate (requires re-priming to maintain)	Configurable based on prompt

“Stability/Consistency” refers to the degree to which the model's personality-like responses remained stable across time, contexts, or repeated measurements, as reported in each study.

However, the stability of these profiles is context- and model-dependent. (Bodroža et al. 2024) found that some models (LLaMA3, GPT-4o) maintain high test-retest reliability, while others (GPT-4, Gemini) show substantial temporal drift. More fundamentally, (Song et al. 2024) demonstrated that a single model (GPT-4) exhibits different inferred personalities when generating original posts versus replying to others—a context sensitivity not observed in humans. Using an external classifier to evaluate open-ended responses, they found that the LLM's “personality” shifted between communicative roles (e.g., ENTJ when posting, INTP when commenting). This finding challenges whether LLMs possess anything analogous to human personality, which entails cross-situational consistency anchored in autobiographical memory and identity.

The controllability of LLM personality also varies markedly. (La Cava and Tagarelli 2025) found that most open models resist persona prompting, maintaining their baseline response patterns even when explicitly instructed to adopt new types. This “closed-mindedness” contrasts with the success of fine-tuning approaches (Cui et al., 2023) and structured prompting frameworks (Besta et al., 2025), which achieve reliable personality conditioning through more intensive intervention. (Cui et al. 2023) created 16 fine-tuned variants, each exhibiting stable behaviors aligned with a specific MBTI type, and reported that these models performed better on tasks congruent with their assigned personality. (Besta et al. 2025) used a prompt-based framework (“MBTI-in-Thoughts”) to temporarily condition off-the-shelf LLMs, finding that self-reflection mechanisms improved cooperation in multi-agent settings and that periodic re-priming helped maintain the desired persona.

Finally, a striking pattern across all studies is the prosocial bias of aligned LLMs. (Bodroža et al. 2024) documented high agreeableness and conscientiousness, low Machiavellianism, and socially desirable responding across models. While this alignment with human values is intended from a safety perspective, it raises questions about whether LLMs can authentically represent the full spectrum of human personality—including less socially desirable but realistic types—for applications such as psychological research or multi-agent simulation. Future work should investigate whether this bias is an inevitable consequence of alignment or can be selectively relaxed under controlled conditions.

### 5.3 Biases and ethical concerns

MBTI-based LLM profiling raises several ethical concerns. First, the risk of reinforcing stereotypes: If an LLM learns that “people who say ‘I love coding' are INTJ” because of dataset quirks, it might systematically misclassify people or, worse, lend undue credibility to pseudoscientific stereotypes (e.g., “INTJs are always antisocial”).

Researchers should carefully audit the features of the models. The retrieval and prototype-based methods help here: by surfacing prototypes or evidence for a decision, one can inspect whether the reasoning is sensible or just biased. For example, ProtoMBTI's prompt-based voting on retrieved prototypes (Li H. et al., 2025) can be analyzed for each trait decision, potentially revealing whether a model thinks that a person is introverted because their post lacked emojis and had formal language—a plausible rationale—or for a spurious reason. Second, privacy: Inferring personality from one's writing can be seen as intrusive, especially if done without consent. Social media users rarely know that every tweet could feed an algorithm guessing their innermost tendencies. If companies deploy LLMs for personality profiling (for targeted advertisements or content moderation), transparency and user control will be vital. Ironically, the popularity of the MBTI could either soften or exacerbate this concern: many people enjoy figuring out their type and might willingly engage, but they might not realize that an AI is doing it behind the scenes on a grand scale.

Regulations on psychometric AI may be needed to prevent misuse (e.g., employers screening candidates via covert personality analysis of their public profiles, a scenario not far-fetched with these models in hand). Furthermore, MBTI is not a valid tool for high-stakes decisions [the U.S. Equal Employment Opportunity Commission has warned against using it in hiring (U.S. Equal Employment Opportunity Commission., 2023)]; therefore, using LLM-derived MBTI assessments in such contexts could be discriminatory or simply ineffective.

### 5.4 MBTI limitations and LLMs

Our discussion would be incomplete without addressing the elephant in the room: the scientific limitations of MBTI. MBTI dichotomies, being bimodal categorizations of continuous traits, suffer from issues such as low test-retest reliability (a person might test as INFP 1 month and ENFP the next if they are near the middle of introversion) and questionable predictive power for real outcomes (as Zárate-Torres and Correa, 2023 found, MBTI types had only a weak link to leadership behavior). Empirical test-retest studies underscore MBTI's instability: in one experiment, only 47% of participants received the same full type classification after five weeks, and individual dimension stability ranged from 61%–78% (Howes and Carskadon, 1979). A comprehensive review by (Pittenger 2005) found that approximately 35%–50% of individuals obtain different classifications on individual dichotomies after four weeks, with meta-analytic evidence confirming mean test-retest correlations of approximately 0.60 over 12-month intervals (Capraro and Capraro, 2002). These establish the reliability ceiling against which LLM accuracies should be interpreted—predicting a construct with 85% accuracy when the construct itself has only 60% stability raises fundamental questions about what is being measured.

LLMs are optimized to reproduce self-reported MBTI labels derived from online corpora. It remains unclear whether models capture underlying trait structure or merely dataset-specific linguistic markers associated with self-reported MBTI labels. For example, intuitive types might explicitly say, “I'm always thinking of possibilities” on forums. An LLM latches onto that phrase as a cue for intuition, which works on the forum, but in general, many intuitive people will not say that outright. Thus, while the MBTI has been a useful benchmark, researchers are considering more robust frameworks. Big Five (OCEAN) traits are one; another interesting direction is the HEXACO model (which extends Big Five with Honesty-Humility)—the MBTI-in-Thoughts authors even mention HEXACO (Besta et al., 2025). It is plausible to combine them: use the MBTI's ease of persona description for the front-end but use Big Five/HEXACO for back-end representation.

Notably, (Besta et al. 2025) show that their approach generalizes beyond the MBTI, implying that one can swap in any trait system.

### 5.5 Interpretability and psychology research

One positive outcome of this interdisciplinary work is the two-way exchange between AI and psychology. Psychologists can use LLMs as tools to test hypotheses about personality and language; for example, does writing style truly reflect MBTI dichotomies? When aligned with psychological theory, as in Proto-MBTI or MBTI-in-Thoughts, LLMs essentially become simulacra for experiments.

For example, with (Besta et al. 2025)'s framework, one could simulate a negotiation between an “ENTJ” and “INFP” agent to see classic MBTI lore in action (ENTJ might dominate the discussion, INFP might seek harmony). If these patterns emerge consistently, they provide a form of validation for personality constructs, albeit *in silico*. Conversely, it might debunk myths—perhaps the INFP-agent actually cooperates fine with ENTJ-agent contrary to stereotype, suggesting context matters more than type. On the AI side, incorporating psychological principles can improve AI design. Prototype theory, as used in ProtoMBTI, provided accuracy and transfer gains (Li H. et al., 2025).

Self-reflection, a concept borrowed from human metacognition, improves agent cooperation. This illustrates that human psychology is a rich source of inductive bias for AI systems. Rather than treating language models as purely engineering artifacts, viewing them through a cognitive science lens (even with something as debated as the MBTI) can inspire novel techniques.

### 5.6 User experience and trust

If an LLM has a detectable personality, users will notice it. Some might prefer one model over another simply because “I vibe with it”—effectively a personality match. Heston's study recommended considering personality in clinical AI deployment (Heston and Gillette, 2025); beyond clinical, any domain involving long-term human-AI interaction should heed this. We might end up with LLM “personality profiles” available to end-users, for example, choosing your AI assistant's personality—do you want a more ESTJ (efficient, no-nonsense) style or an ENFP (enthusiastic, creative) style? Companies have already dabbled in this (some virtual assistants have toggle settings for style/tone, which is a proxy for personality).

The challenge is to do this responsibly and not reinforce users' cognitive biases. People might take an AI's advice more seriously if it sounds confident and authoritative (perhaps a Thinking-Judging type tone), but that does not always correlate with accuracy. Indeed, an agreeable AI might sugarcoat uncertainties, whereas a straightforward analytical AI might bluntly say it does not know the answer. There is no one-size-fits-all solution; context and user needs determine which personality is best. Ideally, an AI could dynamically adjust its approach, but as we saw, most open models are “closed-minded” and cannot fully shape-shift (La Cava and Tagarelli, 2025).

More adaptive models, such as GPT-4, can change their tone with prompting, but even they have intrinsic biases (OpenAI models often come across as over-apologetic and verbose, an artifact of alignment). The prospect of extremely adaptive AI that can impersonate any personality raises the “uncanny valley” of personality: would that feel authentic or disconcerting? Humans value consistency; a system that is bubbly one moment and stoic the next might seem erratic or manipulative to humans.

### 5.7 Toward a unified framework

Perhaps the ultimate goal is not to champion the MBTI or any specific inventory but to arrive at a computational representation of personality that is both grounded in psychology and useful for AI applications. We see pieces of this emerging: embedding-based representations (as in the Maharjan et al., 2025 study, where text embeddings had moderate psychometric validity correlations), multi-trait conditioning (as in MBTI-in-Thoughts allowing Big Five or Enneagram inputs; Besta et al., 2025), and dynamic external evaluation (Song's method to detect persona shifts; Song et al., 2024).

A unified framework might allow an AI to maintain a profile (set of trait values) that is updated slightly with new experiences (to mimic learning and development) but remains bounded for stability. It could then communicate that profile to users in an understandable way (perhaps using MBTI labels because they are user-friendly). Such an AI could also intentionally adjust its profile in certain contexts if advantageous, but with safeguards (like a “core” personality that does not entirely vanish). This is speculative, but it resonates with how humans operate: we all flex our behavior depending on context (work vs. home vs. online), yet we recognize a stable identity under those variations.

### 5.8 Core challenges and future directions

Our analysis reveals three interconnected challenges for MBTI-based personality profiling with LLMs. First, the psychometric limitations of the MBTI construct itself, particularly its low test-retest reliability and forced dichotomization of continuous traits, establish an upper bound on what any predictive model can meaningfully achieve (Pittenger, 2005; Boyle, 1995; Capraro and Capraro, 2002). Second, methodological weaknesses in training data, including self-report biases, domain-specific jargon, and overrepresentation of extreme types, propagate systematic errors into LLM-based systems (Li B. et al., 2025). Third, the philosophical ambiguity surrounding AI personality, specifically whether stable internal dispositions can be attributed to systems whose outputs vary across prompts and versions, fundamentally challenges the analogy between human and machine personality (Song et al., 2024; Bodroža et al., 2024; La Cava and Tagarelli, 2025).

Future work should prioritize: (a) moving toward continuous trait models such as the Big Five or HEXACO, which demonstrate superior psychometric properties and predictive validity (Ozer and Benet-Martinez, 2006; Roberts et al., 2007; Soto, 2019); (b) developing training datasets with soft labels calibrated to population distributions, moving beyond the extreme and self-selected samples that dominate current benchmarks (Li B. et al., 2025); and (c) establishing evaluation frameworks that assess AI personality as context-dependent behavioral tendencies rather than fixed traits, incorporating external evaluation methods and situational judgment tests (Song et al., 2024; Yost et al., 2025). As (Song et al. 2024) argue, “machine ‘personality' should be treated as role- and context-dependent behavioral tendencies rather than a fixed trait,” a perspective that must inform future research design and interpretation.

### 5.9 Closing thoughts

The MBTI and LLMs make for an intriguing combination: one is a famously divisive personality theory, and the other is a transformative technology often seen as a black box. By applying the former to the latter, we gained some clarity (distinct patterns in model behavior) but also confronted deep questions about what it means for a machine to have a personality or understand ours.

Research from 2020–2025 paints a picture of rapid progress, with datasets like MBTIBench raising the bar for realism (Li B. et al., 2025), models like ProtoMBTI pushing accuracy and interpretability (Li H. et al., 2025), and analyses like Heston's and Song's revealing both the presence and fluidity of AI personality (Heston and Gillette, 2025; Song et al., 2024). The field remains methodologically heterogeneous and theoretically unsettled. MBTI-based profiling with LLMs is powerful but double-edged: it can enhance personalization and insight or mislead and oversimplify if uncritically applied.

As we continue to critically analyze and develop this intersection, collaboration between AI experts, psychologists, and ethicists will be essential to ensure that these technologies are used to augment human understanding and not to pigeonhole or manipulate. Ultimately, personality is a rich human concept that we project onto our machines; how we choose to do so will reflect our own values and understanding of ourselves.

## Data Availability

The original contributions presented in the study are included in the article/supplementary material, further inquiries can be directed to the corresponding author.
